# Photocatalytic Degradation of Dissolved Phenol by Immobilized Zinc Oxide Nanoparticles: Batch Studies, Continuous Flow Experiments, and Numerical Modeling

**DOI:** 10.3390/nano12010069

**Published:** 2021-12-28

**Authors:** Michalis V. Karavasilis, Maria A. Theodoropoulou, Christos D. Tsakiroglou

**Affiliations:** 1 Institute of Chemical Engineering Sciences, Foundation for Research and Technology Hellas, Stadiou Street Platani, 26504 Patras, Greece; mkaravas@iceht.forth.gr (M.V.K.); mtheod@iceht.forth.gr (M.A.T.); 2 Department of Chemistry, University of Patras, 26504 Patras, Greece

**Keywords:** photocatalysis, zinc oxide, immobilized nanoparticles, phenol degradation, kinetic constant, parameter estimation

## Abstract

In spite of the progress achieved on the photo-catalytic treatment of water streams, there is still a gap of knowledge on the optimization of the performance of continuous-flow photo-reactors. Zinc-oxide (ZnO) nanoparticles were immobilized on Duranit (80% silica + 20% alumina) inert balls with dip-coating and thermal annealing. The immobilized ZnO nanoparticles were characterized by scanning electron microscopy (SEM), energy dispersive X-ray analysis (EDX), X-ray diffraction (XRD), ultraviolet-visible (UV-Vis) spectroscopy, and Raman spectroscopy. To assess the stability and photocatalytic capacity of immobilized ZnO, degradation tests of phenol were performed in batch mode in a 22 W UV-oven with an emission peak at 375 nm by varying the temperature, the initial phenol concentration, and the ratio of photocatalyst mass to initial phenol mass. Continuous flow tests were conducted on two types of annular photo-reactors, made of poly(methyl)methacrylate (PMMA) and stainless steel (STST), equipped with a 6 W UV-lamp with emission at 375 nm, packed with ZnO-coated Duranit beads. Experiments were conducted by recirculating the phenol solution between the annular space of reactor and an external tank and varying the flow rate and the liquid volume in the tank. A one-dimensional dynamic mathematical model was developed by combining reactive with mass-transfer processes and used to estimate the overall reaction kinetic constant with inverse modeling. The results revealed that the ZnO losses might be discernible in batch mode due to the intense stirring caused by the bubbles of injected air, while an insignificant loss of ZnO mass occurs under continuous flow conditions, even after several cycles of reuse; the order of the overall phenol photodegradation reaction is lower than unity; the pseudo-1st order kinetic constant scales positively with the ratio of photocatalyst mass to the initial phenol mass and Peclet number.

## 1. Introduction

Water is the most valuable good for the proper functioning of the flora and fauna on earth. During the last century, the growing demand for raw materials and energy resources has left residues in the environment and water, which science and industry now has to deal with. Regarding the water resources, there is a great variety of contaminants such as heavy metals, pesticides, organic compounds, bacteria, viruses, etc. [1]. Among them, the phenol and phenolic compounds are considered dangerous due to their extended use, high solubility in water, and strong resistance to complete mineralization. According to Environmental Protection Agency (EPA), phenol can cause irritations to the skin, eyes and mucous membranes after a short-term exposure, and diarrhea, blood, and liver effects after a long-term exposure [2].

The various physical and chemical methods used to remove phenols from wastewater could be classified as either conventional approaches or advanced oxidation processes (AOPs). Adsorption [3], liquid-liquid extraction [4], solid-phase extraction [5], and catalytic wet air oxidation [6] are methods belonging to the first category. On the other hand, advanced oxidation technologies for phenol removal include electrochemical oxidation [7], ozonation [8], Fenton reaction [9], enzymatic treatment [10], and heterogeneous photocatalysis [11,12].

Among the advanced oxidation processes, heterogeneous photocatalysis is the most popular one and has widely been studied as a method for the degradation and removal of hazardous substances from water matrices and gases. The advantage of photocatalysis, compared to other processes, is that no other reagents, except of the photocatalyst and light, are needed. The mechanism behind the photocatalysis is the absorption of light by semiconductor (photocatalyst). This absorption activates the transition of an electron (e−) from the Valence Band (VB) into the Conduction Band (CB), and the simultaneous generation of a positive hole (h+) in the VB. The medium through which the semiconductor comes in contact can lead the carriers (electrons and holes) to a different reaction pathway. Heat can be produced by their recombination, or they can migrate on the semiconductor surface and react with adsorbed molecules through redox reactions. In the case of pollutants dissolved in aqueous medium, e− in the CB can interact with adsorbed oxygen on the surface of photocatalyst by producing a superoxide radical anion (O_2_^–^). Moreover, the h+ in the VB can react with adsorbed water on the semiconductor’s surface by generating hydroxyl radicals (OH) [13].

In heterogeneous photocatalysis, the semiconductor can be suspended inside a reactor or immobilized on a substrate. In recent decades, much emphasis has been placed on photocatalysts suspended in the form of micro- and nanoparticles inside an aqueous medium. In this case, the entire active surface area of photocatalytic particles is exploited, and the rate of pollutant decomposition becomes quite high. However, under continuous flow conditions, it would be necessary to separate the photocatalyst particles from the solution, before reusing them, which is a cost-expensive and energy-intensive step. Alternatively, the photocatalyst particles could be immobilized on various types of substrates, with some examples: clay [14], foams [15,16,17], graphene oxides [18], and borosilicate spheres [19]. The main disadvantage of such an approach is the reduction of photocatalytic efficiency, due to the decrease of the specific surface area, and mass transfer limitations [20,21]. However, these problems can be overcome with the proper design of the photoreactor and the appropriate selection of physicochemical parameters to optimize the photocatalytic activity.

The goal of the present work is to assess the capacity of ZnO nanoparticles immobilized on inert beads to photodegrade organic pollutants in aqueous media. ZnO nanoparticles are immobilized on the surface of Duranit balls, and their photocatalytic activity is tested with experiments of phenol degradation in a batch reactor, placed inside a 22 W UV-oven, and equipped with UV-leds emitting at 375 nm. Continuous flow experiments are performed on two fixed-bed annular photoreactors made of PMMA and stainless steel, respectively, packed with ZnO-coated Duranit balls and illuminated with a cylindrical UV-lamp of 6 W emitting at 375 nm. The aqueous solution of phenol recirculates between each photoreactor and an external vessel able to store variable volume of treated solution. A one-dimensional model, combining the convective flow and hydrodynamic dispersion with overall photodegradation processes, is used for the inverse modelling of experiments and estimation of the kinetic reaction constant as a function of all pertinent parameters [19].

## 2. Materials and Methods

### 2.1. Materials and Chemicals

The following chemicals of analytical grade (Merck) and tri-distilled water were used for the preparation of photocatalysts and solutions: Phenol (C_6_H_6_O), Catechol (C_6_H_6_O_2_) Hydroquinone (C_6_H_6_O_2_), Zinc Acetate dihydrate (ZAC, Zn(CH_3_COO)_2_·2H_2_O), Hydrochloric Acid (HCl) potassium dichromate (K_2_Cr_2_O_7_), sulphuric acid (H_2_SO_4_), Ethanol (95% purity), Sodium Hydroxide (NaOH), Ammonium chloride (NH_4_Cl), Ammonium Hydroxide (NH_4_OH), Potassium ferricyanide (K_3_Fe(CN)_6_), and 4-Aminoantipyrine. Duranit inert balls 3–5 mm (80% SiO_2_–20% Al_2_O_3_) were purchased from VEREINIGTE FÜLLKÖRPER-FABRIKEN-VFF (Baumbach, Germany).

### 2.2. Immobilization of ZnO on Duranit Balls

**Figure 1:**
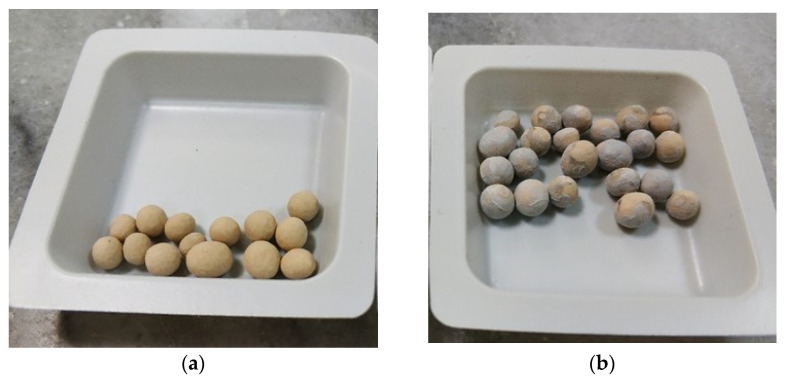
Duranit balls (**a**) before and (**b**) after the ZnO deposition.

The method used to deposit the photocatalyst on the surface of Duranit balls includes dip-coating and thermal annealing. First, an amount of ~10 g of Duranit (Dnit) balls was rinsed with 0.5 M hydrochloric acid, then rinsed with dilute chromosulfuric acid, and finally washed with deionized water and left in an oven at 100 °C until dry. The process of depositing the nanoparticles on the surface of the pure beads consists of the following steps: (a) immersion of the balls in 0.5 M NaOH solution for 30 min at 80 °C; (b) separation of the balls from the solution and placement on a refractory tray; (c) filling the tray with 10 mL 0.3 M zinc acetate precursor solution until covering the Dnit balls; (d) thermal treatment in four successive stages at temperatures 80 °C (solid-solid transformation) [22], 110 °C, 140 °C, and finally 430 °C (annealing), with a duration of 2 h at each stage; (e) removal of ZnO-coated Duranit balls from trays; and (f) rinsing the balls with tri-distilled water to remove ZnO nano-particle aggregates that have not been attached on their surface (Figure 1).

### 2.3. Dnit-ZnO-Photocatalyst Characterization

By removing a sufficient amount of material from the surface of the balls, the photocatalyst was analyzed with XRD on a standard diffractometer (Bruker, D8-Advance, Madison, WI, USA) with Ni- and Co-filtered Cu-Kα1 radiation for crystallinity. A UV–Vis spectrophotometer (UV-1900 Shimadzu, Kyoto, Japan) was used to measure the energy band gap of ZnO nanoparticles, diluted in ethanol at concentration 0.1% *w*/*w*. A SEM Zeiss SUPRA 35VP instrument (Jena, Germany), equipped with an energy dispersive X-ray analysis (EDX) detector, was employed for the optical inspection of the ZnO surface morphology. The surface of the photocatalyst was also analyzed with Raman spectroscopy by using a T64000 Horiba Jobin Yvon micro-Raman setup (Kyoto, Japan), where the 514.5 nm wavelength was emitted from a DPSS laser (Cobolt Fandango TMISO laser, Norfolk, UK) was used for the excitation of the samples. The average thickness of the photocatalyst film was estimated approximately, by weighting the ZnO deposited in several Dnit balls of known mass and diameter.

### 2.4. Dnit-ZnO Photocatalytic Activity

#### 2.4.1. Batch Photocatalysis Tests

**Figure 2:**
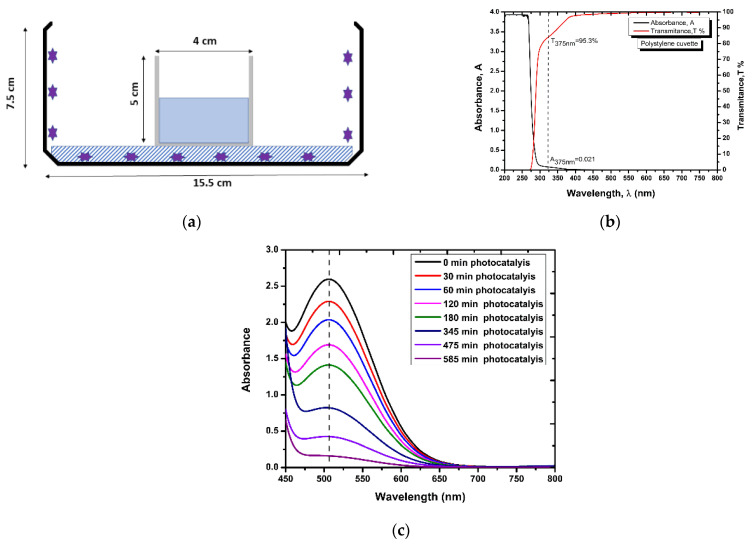
(**a**) Schematic diagram and dimensions of batch photoreactor with the UV-oven of 22 W (**b**) UV-Vis absorption spectrum of polystyrene cuvette. (**c**) UV-Vis spectrum of phenol degradation as a function of time detected by 4-aminoantipyrine method.

Batch photocatalysis tests were conducted in a cuvette made of polystyrene with dimensions 5 cm × 4 cm × 4 cm, thickness 3 mm, and 95.3% permeability to ultraviolet light at the wavelength of 375 nm (Figure 2a,b). ZnO-coated balls of variable mass (~5 g, ~10 g, ~20 g) were placed at the bottom of the cuvette and mixed with 30 mL of phenol solution of variable initial concentration (20, 30, 40 mg/L), whereas atmospheric air was injected at flow rate 1 L/min. All tests were conducted inside a thermostatted incubator (Friocell, Balvanera, Mexico) at three temperatures (20 °C, 25 °C, and 30 °C) and repeated in a second cycle to assess the effects of the ageing and loss of ZnO mass on catalyst performance. The ZnO mass detached from the substrate during the batch tests was measured by weighing the dried ZnO-coated balls before and after the tests.

The aminoantipyrine method [23] was applied to measure the phenol concentration, by collecting occasionally liquid samples and recording the maximum absorption at 505–510 nm in a UV-Vis spectrophotometer (Shimadzu 2700, Figure 2c),

#### 2.4.2. Mechanism of Phenol Degradation

In order to identify the phenol photo-degradation pathways under the prevailing conditions, gas chromatography with flame ionization detection (GC-FID, Shimadzu GC-2014, Kyoto, Japan) was used with a column PTE-5 (Supelco, Bellefonte, PA, USA) of dimensions 30.0 m, 0.25 mm ID, 0.25 μm.

The chromatograms of solutions obtained during the oxidation process were compared with corresponding ones of standard solutions of phenol, catechol, and hydroquinone, which are the main intermediates of phenol oxidation. Liquid/liquid micro-extraction followed: 5 mL of the aqueous solution was collected, its pH was adjusted to 10 by adding 5% *w*/*v* solution of Na_2_CO_3,_ and the solution was centrifuged in 6000 rpm for 5 min; 1 mL of isopropanol and 0.2 mL of dichloromethane were added in the solution and after shaking gently for 10 min, the mixture was centrifuged again to separate the organic from the aqueous phase. 1 μL of the organic phase was injected in the GC-FID. The temperature of the injector, detector, and oven were set to 260 °C, 290 °C, and 50 °C, respectively. The program of column heating included a ramp increase of temperature to 70 °C with a rate of 20 °C/min, holding there for 2 min, and a ramp increase of temperature to 230 °C with rate 15 °C/min, holding there for 2 min.

#### 2.4.3. Continuous Flow Photocatalysis Tests

**Figure 3:**
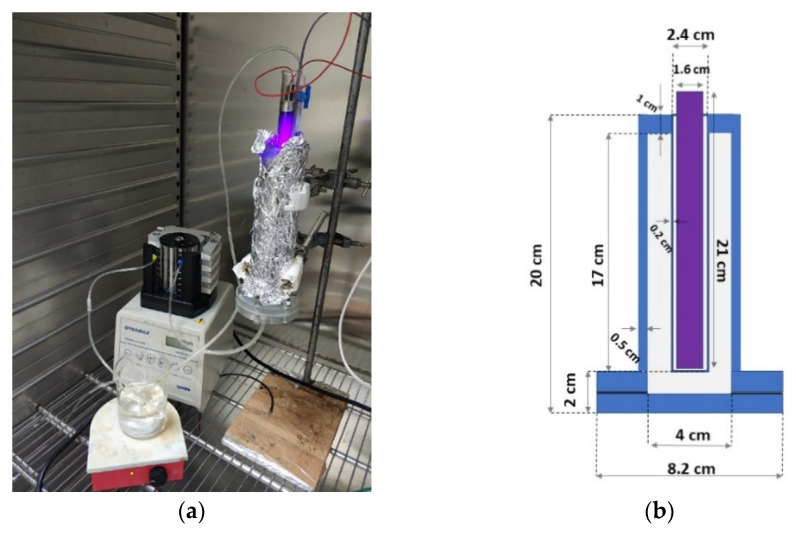
(**a**) Experimental setup of PMMA reactor and (**b**) schematic diagram with dimensions.

**Figure 4:**
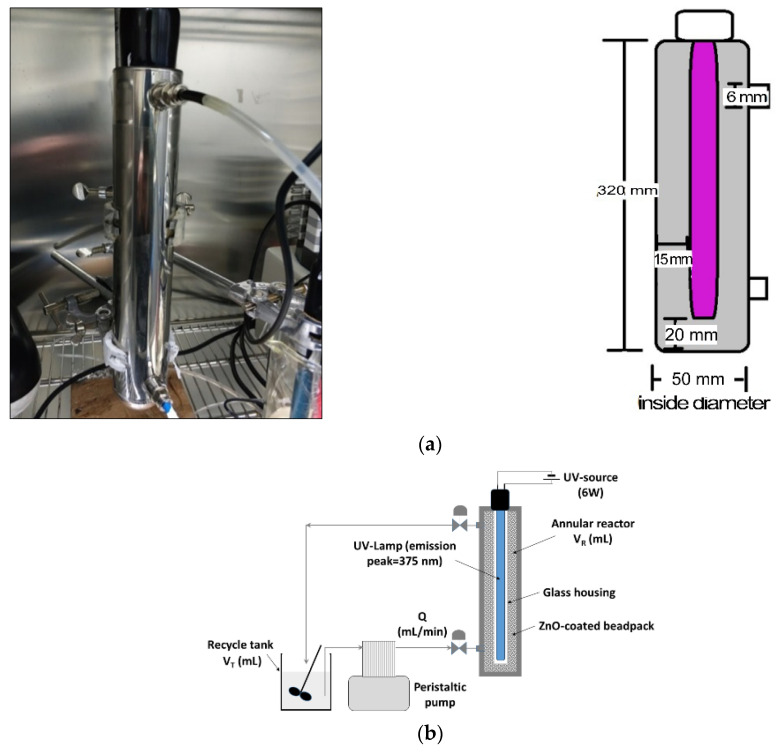
(**a**) Stainless-steel reactor with dimensions. (**b**) Schematic diagram of the experimental setup.

Two fixed-bed annular reactors were used to assess the performance of photocatalysts under continuous flow conditions: one made of poly(methyl) methacrylate (PMMA, Figure 3 [19]) and another one made of stainless steel (STST, Figure 4). The same UV lamp (375 nm Phillips 6 W, Amsterdam, The Netherlands) was placed vertically along the central axis of each reactor and protected by a plastic or glass cover (Figure 4b). The annular space of reactors was packed with ZnO-coated balls: 158.8 g of Dnit supporting 4.3 g of ZnO in a PMMA reactor and 466.8 g of Dnit supporting 8.65 g of ZnO in an STST reactor. The effluent from each reactor was directed to a continuously stirred vessel, from which the solution was fed to the reactor inlet port at a constant flow rate (Figure 3a and Figure 4b) by a peristaltic pump (Rainin, Oakland, CA, USA), ensuring the recirculation of phenol solution. Experiments were conducted at: three values of the dimensionless retention time, $\tau_{TR}$, defined as the retention time in recycle tank to the retention time in fixed-bed reactor (1.75, 3.50, 5.16 for PMMA; 0.22, 1.11, 3.33 for STST); three flow rates (2.0, 10.0 and 50.0 mL/min); a constant initial phenol concentration equal to 20 mg/L. Occasionally, 2 mL of liquid sample was collected from the recycle vessel, and the phenol concentration was measured by UV-Vis spectroscopy. Each experiment was interrupted when the phenol concentration became less than 1 mg/L.

To assess the effect of catalyst ageing on photo-oxidation efficiency, 524.2 g of Dnit balls supporting 6.92 g of ZnO were packed in the STST reactor. Phenol solution of concentration 20 mg/L recirculated at total flow rate of 50 mL/min between the reactor and an external tank of volume 250 mL. The ZnO-coated Dnit balls were used in 5 cycles of photocatalytic degradation.

It is well-known that the wall of PMMA tube, depending on its thickness, absorbs a significant fraction of the UV-radiant flux (80–90%) that has not been absorbed by photocatalyst [19]. On the other hand, the metallic surface of STST reactor is expected to reflect much more efficiently the UV-radiation reaching to it. These two types of photoreactors were selected to elucidate the role of the reactor material and the subsequent losses of the UV-radiant flux on the overall kinetics of phenol oxidation.

To determine the losses of immobilized ZnO, due to its detachment from the substrate, the total mass of the packing material filling in each reactor was weighted. After several cycles of photocatalysis, all balls were removed from each reactor, ZnO was dissolved with chromosulfuric acid, and the balls were dried and weighted again.

#### 2.4.4. Background Experiments

Equilibrium tests of phenol sorption on ZnO surface were conducted at 25 °C by mixing in vials 26 mg of photocatalyst nano-powder with 5 mL of phenol solution of concentrations 1.2, 4.2, 8.1, 10.1, 14.8, 20.0, and 30.0 mg/L and placing the vials in a rotator for 24 h.

To examine whether phenol is oxidized when irradiated by UV-light at 375 nm, a photolysis experiment without catalyst was performed in batch mode at 25 °C, by placing 100 mL of phenol solution at concentration 31.5 mg/L in the cuvette inside the UV-oven and injecting air at flow rate 50 mL/min.

#### 2.4.5. Mathematical Modeling and Numerical Simulation

The mass balance of phenol around the photoreactor is described by the advection-dispersion-reaction equation:

(1) $$\frac{\partial C_{R}}{\partial t}=D_{L}\frac{\partial^{2}C_{R}}{\partial x^{2}}-u_{0}\frac{\partial C_{R}}{\partial x}-r_{dis}$$

where t is the time, $C_{R}\left(t,z\right)$ is the phenol concentration along the photoreactor, x is the axial distance from the inlet of the column, $D_{L}$ is the longitudinal hydrodynamic dispersion coefficient, $u_{0}=Q/\left(\varphi A\right)$ is the average pore velocity, φ is the bed porosity, Q is the volumetric flow rate, A is the cross-sectional area of the bed, $r_{dis}$ is the overall reaction rate of dissolved phenol, and $C_{Τ}\left(t\right)$ is the phenol concentration in the recycle tank (Figure 4b). The mass balance of phenol around the recycle vessel, regarded as a continuously stirred tank:

(2) $$\frac{dC_{T}}{dt}=\frac{Q}{V_{T}}\left[C_{R}\left(t,z=L\right)-C_{T}\right]$$

where $V_{Τ}$ is the liquid volume in the tank. The overall rate of the photocatalytic reaction, regarded as homogeneous process, can be described by pseudo-first order kinetics of the form:

(3) $$r_{dis}=k_{r}C_{R}$$

where $V_{R}$ is the volume of the annular space of reactor (Figure 4b), and $k_{r}$ is the kinetic constant (s^−1^). The hydrodynamic dispersion coefficient is given by the following [19]:

(4) $$D_{L}=\frac{D_{m}}{F\varphi }+a_{L}u_{0}$$

where $D_{m}$ is the molecular diffusion coefficient, $a_{L}$ is the longitudinal dispersion length, and F is the electrical formation factor. If $C_{0}$ is the initial phenol concentration, then, using the dimensionless variables, $\tau =tu_{0}/L$, ξ=x/L, $C_{R}^{*}=C_{R}/C_{0}$, and $C_{T}^{*}=C_{T}/C_{0}$, we get the following system of dimensionless parametric equations

(5) $$\frac{\partial C_{R}^{*}}{\partial \tau }=\left[\left(\frac{D_{m}}{u_{0}L}\right)\frac{1}{F\varphi }+\frac{a_{L}}{L}\right]\frac{\partial^{2}C_{R}^{*}}{\partial \xi^{2}}-\frac{\partial C_{R}^{*}}{\partial \xi }-\left(\frac{k_{r}L}{u_{0}}\right)C_{R}^{*}$$

(6) $$\frac{dC_{T}^{*}}{d\tau }=\left(\frac{\varphi V_{R}}{V_{T}}\right)\left[C_{R}^{*}\left(\tau ,\xi =1\right)-C_{T}^{*}\right]$$

which are coupled with the initial conditions

(7) $$C_{R}^{*}\left(\tau =0,\xi \right)=1.0$$

(8) $$C_{Τ}^{*}\left(\tau =0\right)=1.0$$

and boundary conditions

(9) $$C_{R}^{*}\left(\tau ,\xi =0\right)=C_{T}^{*}\left(\tau \right)$$

(10) $$\frac{\partial C_{R}^{*}}{\partial \xi }\left(\tau ,\xi =1\right)=0$$

The following set of dimensionless parameters are included, explicitly or implicitly, into Equations (5) and (6) [19]:

Peclet number, $Pe=\frac{u_{0}L}{D_{m}}$

Damköhler number, $Da=\frac{Lk_{r}}{u_{0}}$

Dimesnionless retention time, $\tau_{TR}=\frac{V_{T}}{\varphi V_{R}}$

Dimensionless dispersion length, $a_{L}^{*}=\frac{a_{L}}{L}$

Dimensionless catalyst mass, $M=\frac{W_{c}}{\varphi V_{R}C_{0}}$

By using as input data the geometrical characteristics of photoreactors, the solution volume in recycle tank, and the experimental conditions (Table 1), the foregoing system of equations was solved in the platform of ATHENA Visual Studio 14 software [24] and its numerical solution was fitted to the transient measurements of phenol concentration in the recycle tank to estimate the kinetic constant of the pseudo-first order photocatalytic reaction, $k_{r}$.

## 3. Results and Discussion

### 3.1. Morphological and Optical Properties

**Figure 5:**
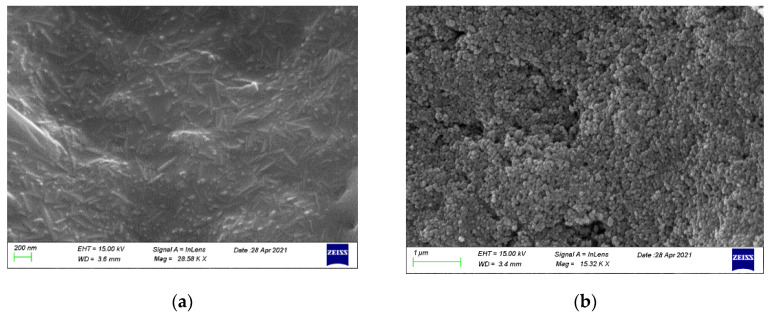

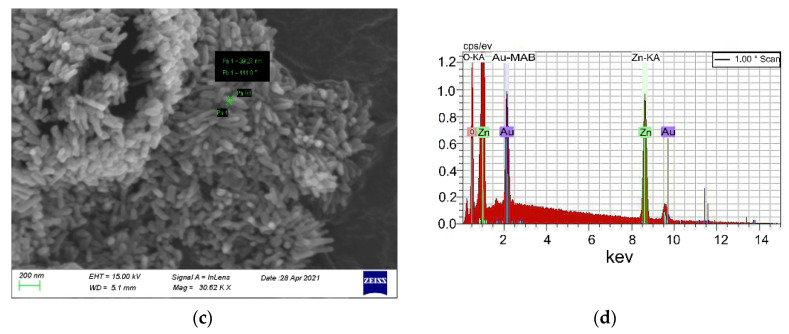
Scanning electron microscopy (SEM) images of the Duranit balls surface (**a**) before, and (**b**,**c**) after the ZnO deposition. (**d**) EDX spectrum of the Duranit balls surface after the deposition.

The surface of Duranit balls before (Figure 5a) and after (Figure 5b,c) the ZnO deposition is shown on SEM images. It is covered almost fully by a uniform and dense layer of ZnO nanorods of diameter ~37–50 nm, and length ~150–250 nm, in agreement with earlier work [19].

The EDX spectrum (Figure 5d) shows the presence of zinc and oxygen on the surface of the beads, thus identifying the ZnO oxide composition.

**Figure 6:**
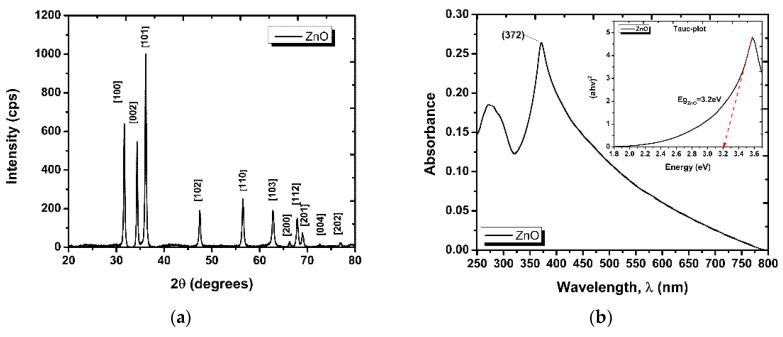

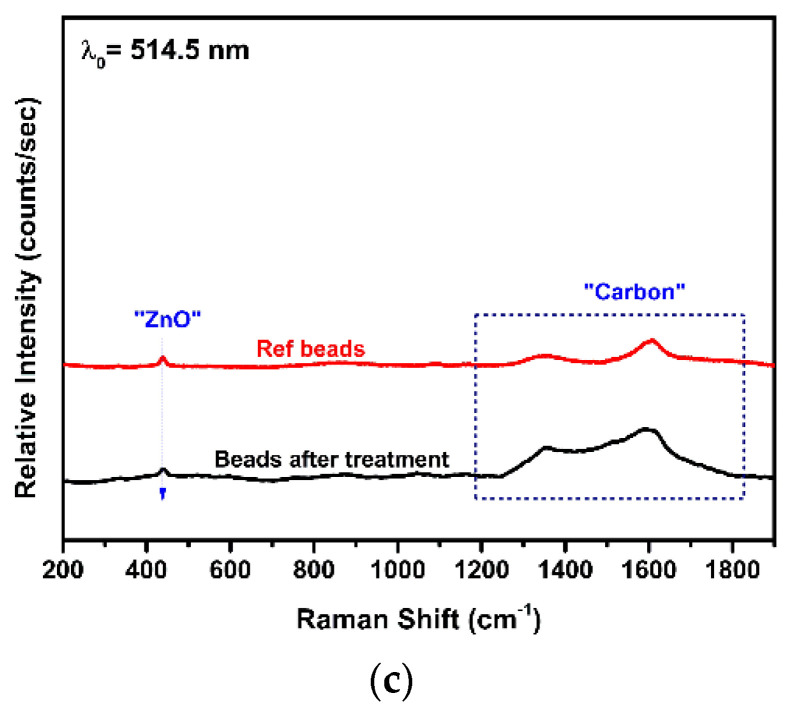
(**a**) XRD pattern of ZnO powder. (**b**) UV-Vis spectrum of a suspension of ZnO nanoparticles with the application of Tauc-plot method (inset). (**c**) Raman spectra of ZnO-coated balls.

Regarding the XRD analysis, the diffraction patterns were collected over 2θ range ~20–80°. The phase composition was determined by using the phase analysis software Match!, version v3.12 (Crystal Impact GbR, Bonn, Germany). The characteristic peaks of pure ZnO (Zincite) were observed (Figure 6a). The sharp intense peaks of ZnO confirmed the high crystalline structure of the sample, whereas the peaks (100), (002), (101), (102), (110), (103), (200), (112), (201), (004), and (202) refer to the hexagonal wurtzite structure of ZnO and confirmed the success of the synthesis (Figure 6a).

The characteristic peak of the hexagonal wurtzite appears at wavelength 372 nm on the UV-Vis spectrum of ZnO suspension (Figure 6b), from which an energy band gap ~3.2 eV was determined with the Tauc-plot method [25].

In the Raman spectrum (Figure 6c), the peak at 438 cm^−1^ corresponds to the *E*2 mode, typical of the hexagonal phase of ZnO [26]. The peaks at 1350–1650 cm^−1^ are most likely attributed to D and G bands of carbon residues of zinc acetate that might have remained on the surface after the thermal treatment [27].

Accounting for the density of ZnO (ρ_ZnO_ = 5.61 g/cm^3^), the actually measured diameter of the balls (3–6 mm), the density of Duranit balls (ρ_Dnit_ = 2.3 g/cm^3^), and assuming that the fraction of surface coverage with ZnO nanoparticles changes from 0.8 to 1.0, the thickness of the deposited ZnO layer was estimated to be ~4–5 μm.

### 3.2. Batch Tests of Photocatalysis

#### 3.2.1. Sorption of Phenol

**Figure 7:**
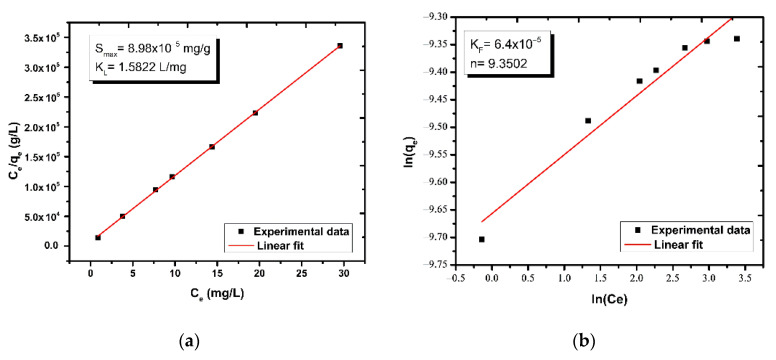
Linear fitting of (**a**) Langmuir and (**b**) Freundlich models.

Equilibrium sorption data from tests performed in the dark were fitted to Langmuir isotherm, Equation (11), and Freundlich isotherm, Equation (12), to estimate all pertinent parameters $K_{L},q_{max}$ (Figure 7a) and $K_{F},n$ (Figure 7b), respectively. It is evident that the maximum phenol concentration adsorbed on the ZnO surface at equilibrium was quite low (~10^−4^ mg/g), and for this reason the phenol adsorption was overlooked in subsequent analysis.

(11) $$q_{e}=\frac{K_{L}q_{max}C_{e}}{1+K_{L}C_{e}}$$

(12) $$q_{e}=K_{F}C_{e}$$

#### 3.2.2. Photolysis

**Figure 8:**
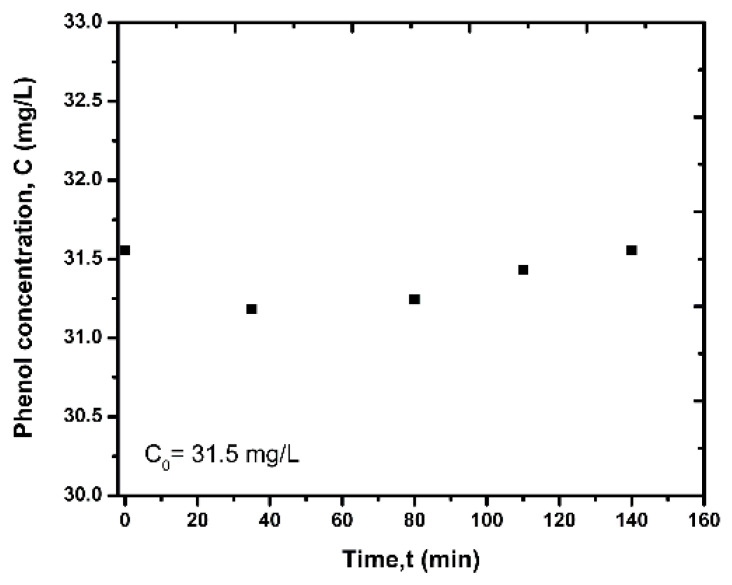
Transient response of phenol concentration during photolysis test.

The transient photolysis response of phenol is shown in Figure 8. As shown, the effect of ultraviolet radiation does not significantly affect the initial concentration of phenol, and its oxidation is negligible.

#### 3.2.3. Parametric Analysis of Batch Tests

The mass of deposited ZnO per unit of mass of substrate in the 1st and 2nd cycle of photo-degradation tests is shown in Table 2 and Table 3, respectively.

**Figure 9:**
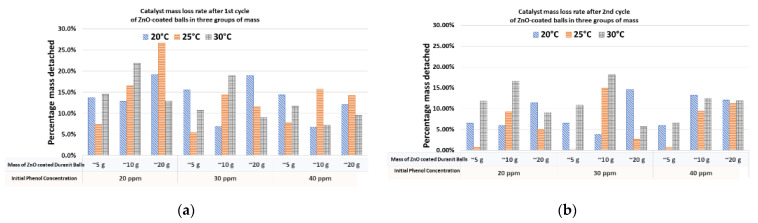
The percentage of ZnO mass detached from Duranit balls after the (**a**) 1st and (**b**) 2nd cycle of photocatalysis.

The leaching of catalyst mass after each cycle is illustrated in Figure 9. It seems that a significant percentage of the deposited ZnO mass, ranging from 5 to 25%, is leached to the liquid phase during the 1st cycle of photocatalysis tests (Figure 9a), while the ZnO leaching weakens during the 2nd cycle of photocatalysis tests (Figure 9b). The main reason for the leaching is the intense stirring caused by the air bubbles due to the high flow rate of injected air and has been analyzed in extent elsewhere [19].

**Figure 10:**
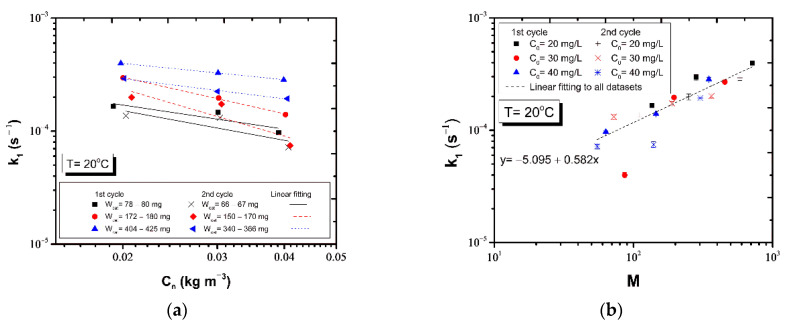

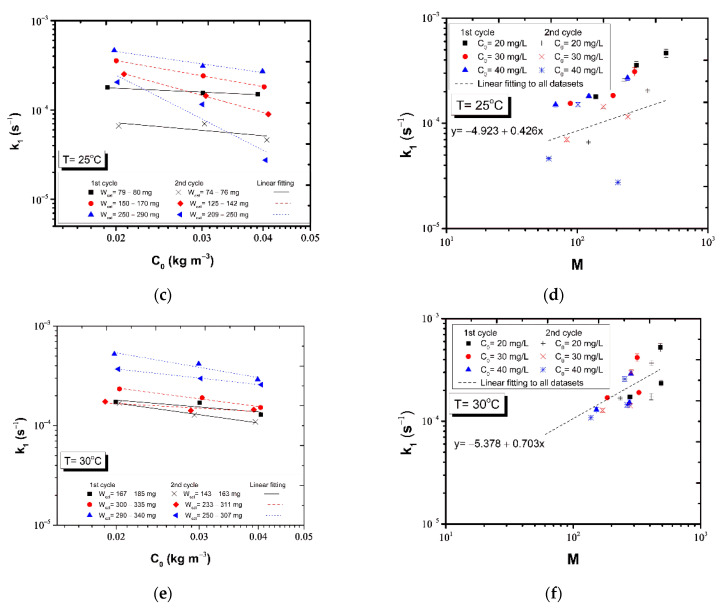
Plots of the apparent first order rate constant vs. the initial phenol concentration at temperature (**a**) 20 °C (**c**) 25 °C (**e**) 30 °C. Plots of the apparent first order kinetic constants vs. the ratio M at temperature (**b**) 20 °C (**d**) 25 °C (**f**) 30 °C.

It is evident that the kinetic constant decreases weakly when the photocatalyst is reused and some of its mass has been detached, while no significant effect of the temperature on $k_{1}$ value was realized (Figure 10). A linear regression analysis was conducted for the pseudo-first order kinetic constants estimated from tests conducted at each temperature with regard to the initial phenol concentration (Figure 10a,c,e) and ratio M of catalyst mass to the initial phenol mass (Figure 10b,d,f). The results of k_1_ vs. C_0_ were classified into datasets according to the initial mass of photocatalyst and cycle of photocatalysis and plotted on a log scale (Figure 10a,c,e). The slope of the linear regression can be used as a quantitative criterion to assess the distance of the overall reaction rate from a 1st order process. Approximately, the order m of the overall process could be extracted from the exponent of the scaling law

(13) $$k_{1}\propto C_{0}{}^{m-1}$$

Given that m−1<0 (Table 4, Figure 10a,c,e), it turns out that m<1, namely that the overall photocatalytic reaction deviates from a 1st order process.

Regardless of the catalyst mass and cycle of photo-degradation tests, all datasets obtained at each temperature were used to examine the potential correlation of the kinetic constant with the ratio M (Figure 10b,d,f). In all cases, it is evident that k_1_ is an increasing function of M, and scales as

(14) $$k_{1}\propto M^{a}$$

with the exponent a ranging between 0.4 and 0.7 (Figure 10b,d,f).

#### 3.2.4. Mechanism of Phenol Degradation

**Figure 11:**
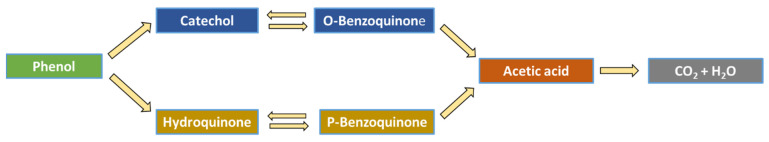
General mechanism of phenol photocatalytic degradation.

A general pathway of phenol degradation during photocatalysis is shown in Figure 11 [28,29] and is explained below.

**Figure 12:**
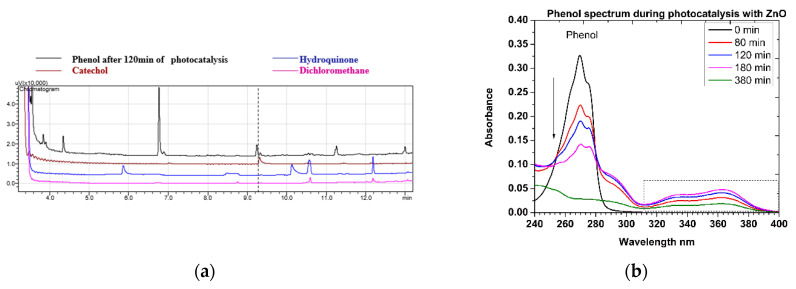
(**a**) GC-FID chromatogram of phenol degradation products extracted in dichloromethane, after 120 min of photocatalytic reaction as compared with the corresponding ones of catechol, hydroquinone, and dichloromethane (solvent). (**b**) Spectrum of the UV-Vis absorbance of phenol degradation products as a function of time.

In the GC-FID chromatograph (Figure 12a), a characteristic peak of phenol appears at 6.9 min and a peak at 9.2 min, which identifies the catechol, namely the upper pathway of the mechanism (Figure 11), while the characteristic peak of hydroquinone at 5.9 min is not evident. Theoretically, the probability for the formation of catechol (ortho-position) is two-fold than the probability for the formation of hydroquinone (para-position), and therefore it is reasonable to identify only the catechol at advanced stages of the photo-degradation.

The gradual photocatalytic degradation of phenol to other products is evident at the wavelength of 269 nm over the UV absorbance spectrum (Figure 12b). However, at the same time, a bimodal curve with maxima at 325 nm and 370 nm starts strengthening and then weakening. The transient variation of this bimodal curve is indicative of the presence of o- and p-benzoquinones (Figure 11), the degradation of which signifies the relatively slow step of the phenol degradation pathway, until progressively both disappearing [30,31].

### 3.3. Continuous Flow Tests of Photocatalysis

The inverse modeling of continuous flow tests, conducted with the two types of reactors, allows us to estimate the overall reaction kinetics and correlate it with dimensionless parameter values, whereas the results are summarized in Table 5. Accounting for the variable initial mass of phenol in recycle tank (Figure 4b), an additional parameter that expresses the ratio of catalyst mass to the total initial mass of pollutant, $M^{\prime }$, is defined by:

(15) $$M^{\prime }=\frac{M}{1+\tau_{TR}}$$

**Figure A1:**
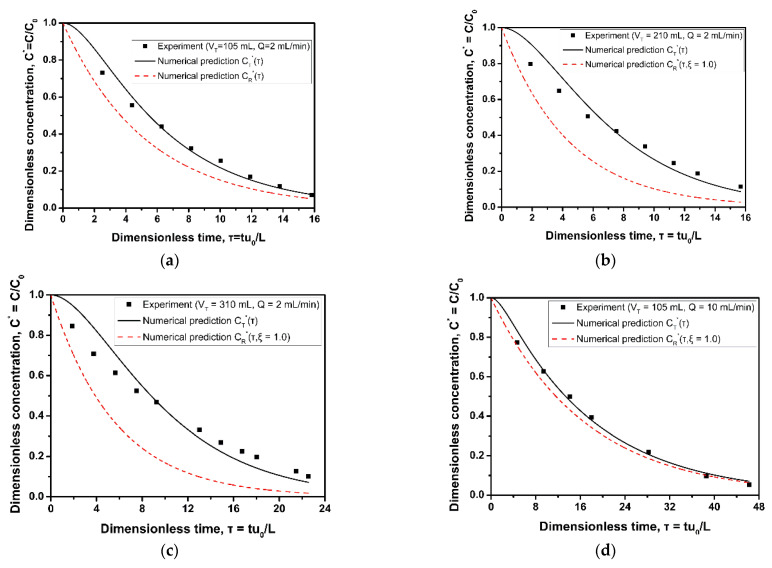

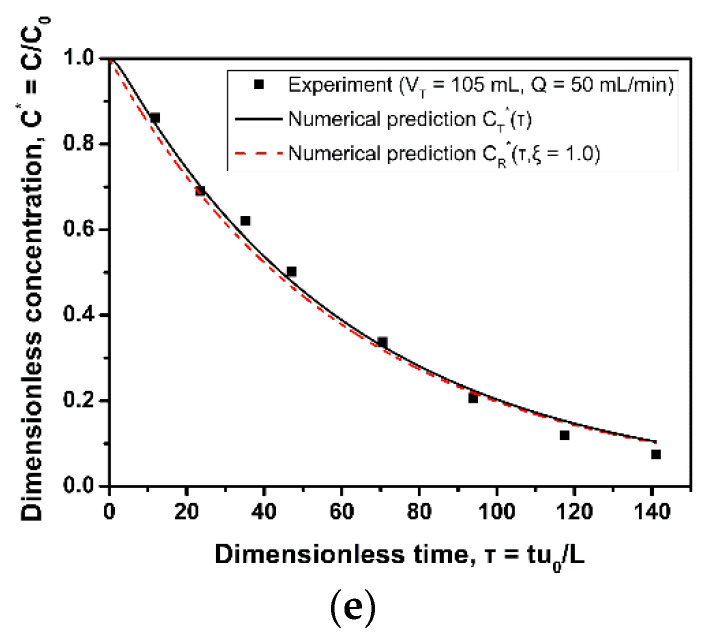
Comparison of numerical prediction with experimental measurements of the transient response of phenol concentration in continuous flow PMMA photoreactor for various values of the flow rate and liquid volume in recycle tank. (**a**) V_T_ = 105 mL, Q = 2 mL/min; (**b**) V_T_ = 210 mL, Q = 2 mL/min; (**c**) V_T_ = 310 mL, Q = 2 mL/min; (**d**) V_T_ = 105 mL, Q = 10 mL/min; (**e**) V_T_ = 105 mL, Q = 50 mL/min.

**Figure A2:**
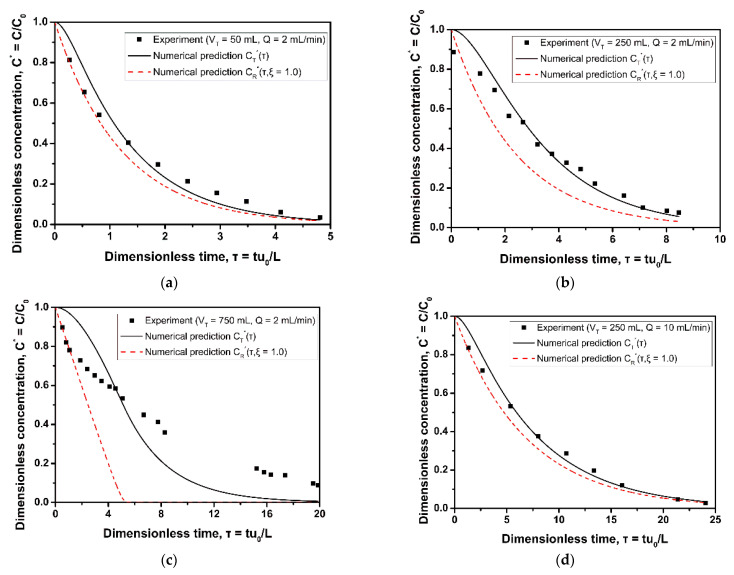

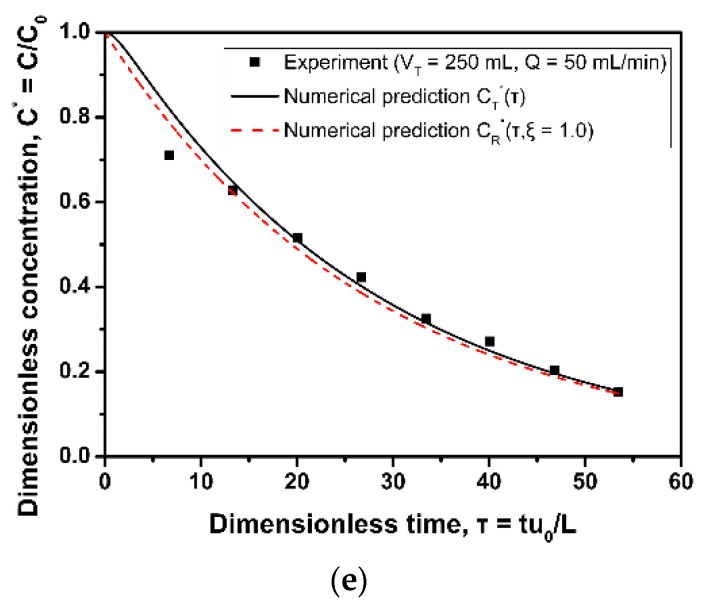
Comparison of numerical prediction with experimental measurements of the transient response of phenol concentration in continuous flow STST photoreactor for various values of the flow rate and liquid volume in recycle tank. (**a**) V_T_ = 50 mL, Q = 2 mL/min; (**b**) V_T_ = 250 mL, Q = 2 mL/min; (**c**) V_T_ = 750 mL, Q = 2 mL/min; (**d**) V_T_ = 250 mL, Q = 10 mL/min; (**e**) V_T_ = 250 mL, Q = 50 mL/min.

The transient response of measured phenol concentration was predicted satisfactorily by the model, over the majority of parameter values, for both the PMMA (Appendix A, Figure A1a,b,d,e) and STST (Appendix A, Figure A2a,b,d,e) reactors. Discrepancies between experiment and prediction were observed mainly at the lowest flow rate and maximum liquid volume in recycle tank (Figure A1c and Figure A2c). Under such conditions, it seems that the performance of the reactor is slower than that predicted (Figure A1c and Figure A2c), and the discrepancy might be attributed to: (i) the insufficient (reacting) retention time in photocatalytic reactor compared to the long (non-reacting) retention time in recycle tank; (ii) non-uniformities of the flow field across the annular space and enhancing its deviation from the fully developed and one-dimensional flow. Evidently, $k_{r}$, estimated from continuous flow tests (Table 5) is comparable to the corresponding values of $k_{1}$, estimated from batch tests (Figure 10). Additionally, all $k_{r}$ values are of the same order of magnitude regardless of the reactor type (Table 5) supposing that all other properties (e.g., geometric dimensions, catalyst mass per unit mass of substrate) are overlooked.

**Figure 13:**
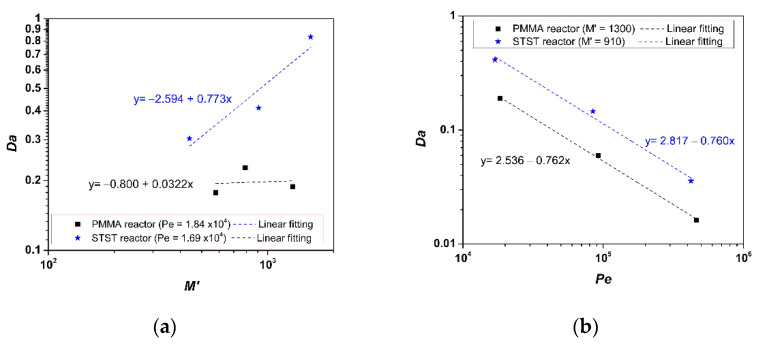
Dependence of Damköhler number on (**a**) the ratio of catalyst mass to the initial mass of pollutant and (**b**) the Peclet number.

Moreover, it seems that $k_{r}$ is an increasing function of the Peclet number and an increasing function of $M^{\prime }$ for the STST reactor, but almost independent of $M^{\prime }$ for PMMA reactor (Table 5). At progressively increasing Pe values, the higher pore velocities result in thinner boundary layers of dissolved phenol concentration surrounding the grains, thus increasing the phenol concentration gradient and decreasing the resistance to the mass transfer from the bulk to the catalyst surface, which is reflected in higher overall $k_{r}\;$ values (Table 5). Though $k_{r}$ is a purely increasing function of $M^{\prime }$ for STST (Figure 13a), it becomes almost independent of $M^{\prime }$ for PMMA at high values of this parameter (Figure 13a). This might be associated with the less UV-radiant flux that is available to photocatalyst absorption in the PMMA reactor due to the UV-light absorption by the PMMA housing of UV-lamp and external PMMA wall [19].

For both reactors, the scaling law $Da\propto Pe^{-0.76}$ (Figure 13b) is obtained. Given that $Da\propto k_{r}/Pe$, it turns out the power law $k_{r}\propto Pe^{0.24}$, which is a significant relationship that could be used to scale-up the performance of photoreactors.

The mass balance of ZnO-coated Dnit packing before and after the photocatalytic tests revealed that the losses of deposited ZnO were 2.4% for the PMMA reactor, and 4.7% for the STST reactor, both being much less than the losses observed in batch reactors [19].

**Figure 14:**
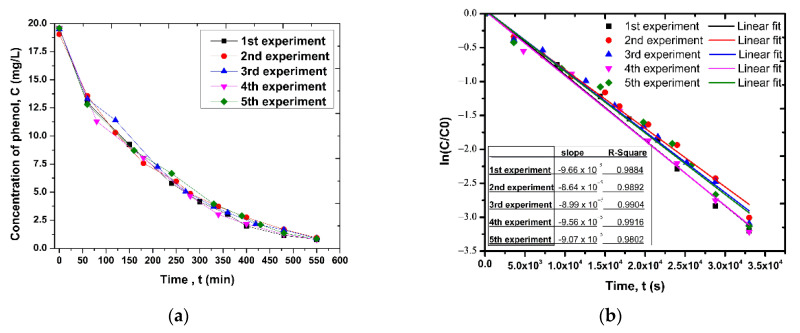
(**a**) Phenol concentration in recycle tank as a function of time for five cycles of photocatalysis, performed on a STST reactor with the same pack of ZnO-coated Dnit balls. (**b**) Calculation of the slope of the curves.

The ageing of photocatalysts was tested in an STST reactor by setting the flow rate at 50 mL/min and phenol solution volume in a recycle tank equal to 250 mL. It was found that the photocatalyst continued to be efficient after five (5) cycles with very weak changes of the transient phenol concentration response from cycle to cycle, which is reflected to an almost identical kinetic constant for the overall phenol oxidation process (Figure 14).

## 4. Conclusions

ZnO nanoparticles were immobilized on Duranit balls, and their photocatalytic activity was tested with phenol photo-degradation tests. Batch tests were conducted in a polystyrene cuvette illuminated by a 22 W UV-oven emitting at 375 nm, and continuous flow tests were performed on two fixed-bed annular photoreactors made of PMMA and STST, connected with an external recycle vessel, and illuminated by a 6 W UV-lamp emitting at 375 nm. A one-dimensional model, combining the convective flow and hydrodynamic dispersion with the overall photodegradation reaction, was used for the inverse modelling of experiments and estimation of kinetic reaction constant as a function of all pertinent parameters. The most important conclusions are outlined below:

- ZnO nanorods of diameter 38–50 nm, length 0.15–0.25 μm were attached on the surface of Duranit balls by creating uniform coatings of thickness 4–5 μm.
- ZnO nanoparticles immobilized on Duranit beads were proven very efficient toward the photodegradation of phenol.
- Significant detachment of ZnO might occur under the intense stirring caused by injected air, while the loss of immobilized ZnO is insignificant under continuous flow conditions.
- Comparable values of kinetic constant were obtained from tests conducted in batch and continuous modes.
- The immobilized ZnO photocatalysts maintain their performance even after their reuse for several cycles.
- The overall phenol photo-degradation process on immobilized ZnO catalyst is an m-order process with m < 1.
- The phenol photo-degradation mechanism agrees with the general scheme of two parallel pathways with catechol and hydroquinone as intermediates transformed into ortho- and para- benzoquinones, the oxidation of which is quite slow.
- The pseudo-first order kinetic constant resulting from batch tests scales with the ratio of catalyst mass to initial phenol mass, M, according to a power law with an exponent between 0.4 and 0.7, depending on the temperature and catalyst ageing.
- The pseudo-first order kinetic constant resulting from continuous flow tests scales with the Peclet number according to a power law with an exponent equal to 0.24.

## Figures and Tables

**Table 1:** Geometrical properties of fixed-bed photoreactors and experimental conditions.

Property	PMMA	STST
φ	0.42	0.42
A (m^2^)	8.042 × 10^−4^	1.649 × 10^−3^
L (m)	0.189	0.324
$d_{g}$ (mm)	3.0–5.0	3.0–5.0
m	1.5	1.5
$a_{L}$ (m)	1.0 × 10^−2^	1.0 × 10^−2^
$D_{m}$ (m^2^ s^−1^)	9.1 × 10^−10^	9.1 × 10^−10^
$C_{0}$ (mg/L)	20.0	20.0
W_cat_ (g)	4.306	8.645
W_subs_ (g)	158.77	467.19
Q (mL/min)	2, 10, 50	2, 10, 50
$V_{T}$ (mL)	105, 210, 310	50, 250, 750

**Table 2:** 1st cycle of photo-degradation batch tests.

Initial Phenol Concentration		20 mg/L			30 mg/L			40 mg/L		
		Mass of Deposited ZnO Photocatalyst (mg)								
Mass of Duranit Balls (g)		~5	~10	~20	~5	~10	~20	~5	~10	~20
Temperature	20 °C	78	172	425	78	182	404	78	182	416
	25 °C	80	170	285	80	170	248	80	148	290
	30 °C	167	299	287	167	299	285	185	335	340

**Table 3:** 2nd cycle of photo-degradation batch tests.

Initial Phenol Concentration		20 mg/L			30 mg/L			40 mg/L		
		Mass of Deposited ZnO Photocatalyst (mg)								
Mass of Duranit balls (g)		~5	~10	~20	~5	~10	~20	~5	~10	~20
Temperature	20 °C	67.3	149.8	343.3	65.9	169.3	327.3	66.8	169.8	366
	25 °C	74.1	142	208.7	75.7	145.5	219.4	73.8	124.6	248.7
	30 °C	142.7	233.4	249.7	149.1	242.2	259.3	163.4	311	307.4

**Table 4:** Summary of the slopes obtained from linear regression analysis of batch tests.

	20 °C		25 °C		30 °C	
Cycle	Catalyst Mass (mg)	m–1	Catalyst Mass (mg)	m–1	Catalyst Mass (mg)	m–1
1st	78–80	–0.70724	79–80	−0.24838	167–185	−0.39258
2nd	66–67	−0.87037	74–76	−0.48008	143–163	−0.66167
1st	172–182	−1.06729	150–170	−0.96533	300–335	−0.61697
2nd	150–170	−1.3849	125–142	−1.51594	233–311	−0.26766
1st	404–425	−0.47856	250–290	−0.7849	290–340	−0.82332
2nd	340–366	−0.59051	209–250	−2.79594	250–307	−0.51947

**Table 5:** Dimensionless parameters of flow-through experiments and estimated values of reaction kinetic constant.

Reactor Type	Q (mL/min)	*V_T_* (mL)	*M*	*τ_TR_*	*M’*	*Pe*	Kinetic Constant, *k_r_* (s^−1^)	*Da*
PMMA	2.0	105	3.59 × 10^3^	1.75	1.30 × 10^3^	1.84 × 10^4^	(0.985 ± 0.111) × 10^−4^	0.1886
	2.0	210		3.50	0.79 × 10^3^	1.84 × 10^4^	(1.189 ± 0.249) × 10^−4^	0.2277
	2.0	310		5.16	0.58 × 10^3^	1.84 × 10^4^	(0.929 ± 0.218) × 10^−4^	0.1779
	10.0	105		1.75	1.30 × 10^3^	9.22 × 10^4^	(1.555 ± 0.143) × 10^−4^	0.0595
	50.0	105		1.75	1.30 × 10^3^	46.1 × 10^4^	(2.115 ± 0.159) × 10^−4^	0.0162
STST	2.0	50	1.92 × 10^3^	0.22	1.57 × 10^3^	1.69 × 10^4^	(1.239 ± 0.239) × 10^−4^	0.834
	2.0	250		1.11	0.91 × 10^3^	1.69 × 10^4^	(0.612 ± 0.075) × 10^−4^	0.412
	2.0	750		3.33	0.44 × 10^3^	1.69 × 10^4^	(0.452 ± 0.144) × 10^−4^	0.305
	10.0	250		1.11	0.91 × 10^3^	8.46 × 10^4^	(1.087 ± 0.147) × 10^−4^	0.146
	50.0	250		1.11	0.91 × 10^3^	42.3 × 10^4^	(1.324 ± 0.153) × 10^−4^	0.035

## Data Availability

The data is available on reasonable request from the corresponding author.

## Appendix A

A comparative analysis of inverse modeling numerical predictions with experimental datasets of tests performed on a continuous flow photoreactors is shown in Figure A1 and Figure A2.
